# Socioeconomic Inequalities in Elective and Nonelective Hospitalizations in Older Men

**DOI:** 10.1001/jamanetworkopen.2022.6398

**Published:** 2022-04-07

**Authors:** Peiyao Xu, Fiona M. Blyth, Vasi Naganathan, Robert G. Cumming, David J. Handelsman, Markus J. Seibel, David G. Le Couteur, Louise M. Waite, Saman Khalatbari-Soltani

**Affiliations:** 1 Faculty of Medicine and Health, The University of Sydney School of Public Health, Sydney, New South Wales, Australia; 2 ARC Centre of Excellence in Population Aging Research (CEPAR), University of Sydney, Sydney, New South Wales, Australia; 3 Concord Clinical School, Faculty of Medicine and Health, University of Sydney, Sydney, New South Wales, Australia; 4 Centre for Education and Research on Ageing, Faculty of Medicine and Health, University of Sydney, Sydney, New South Wales, Australia; 5 Ageing and Alzheimer’s Institute, Concord Repatriation and General Hospital, Sydney Local Health District, Concord, New South Wales, Australia; 6 ANZAC Research Institute, University of Sydney and Concord Hospital, Sydney, New South Wales, Australia

## Abstract

**Importance:**

Among older adults, there is limited and inconsistent evidence on the association between socioeconomic position (SEP) and elective and nonelective hospitalization.

**Objective:**

To evaluate the association between SEP and all-cause and cause-specific elective and nonelective hospitalization and hospital length of stay among older men.

**Design, Setting, and Participants:**

This population-based, prospective cohort study used data from the Concord Health and Aging in Men Project (CHAMP). CHAMP recruited 1705 men aged 70 years or older between January 28, 2005, and June 4, 2007, in Sydney, Australia. Data were analyzed from February 1 to September 30, 2021.

**Exposures:**

Indicators of SEP, including education (university degree certificate, diploma or no postschool qualifications), occupation (professionals and managers; small employers and self-employed; or lower clerical, service, sales workers, skilled, and unskilled workers), and source of income (other sources of income than government pension, reliance on government pensions and other sources of income, or reliant solely on a government pension), and a cumulative SEP score (tertiles) as SEP indicators; 3-level variables present high, intermediate, and low SEP.

**Main Outcomes and Measures:**

All-cause and cause-specific elective and nonelective hospitalizations, number of hospitalizations, and length of stay were the study outcomes, ascertained through data linkage. Associations were quantified using competing-risks survival regression and negative binomial regression.

**Results:**

A total of 1566 men (mean [SD] age, 76.8 [5.4] years) were included. During a mean (SD) 9.07 (3.53) years of follow-up, 1067 men had at least 1 elective hospitalization, and 1255 men had at least 1 nonelective hospitalization. No associations were found between SEP and elective hospitalizations. Being in the lowest tertile for educational level (subhazard ratio [SHR], 1.32; 95% CI, 1.11-1.58), occupational position (SHR, 1.30; 95% CI, 1.12-1.50), sources of income (SHR, 1.33; 95% CI, 1.17-1.52), and cumulative SEP tertile groups (SHR, 1.45; 95% CI, 1.24-1.68) were all associated with having at least 1 nonelective hospitalization compared with those in the highest tertiles. Significant associations were found between being in the lowest SEP groups and increased numbers and longer length of stay of nonelective hospitalizations.

**Conclusions and Relevance:**

In this prospective cohort study, low SEP was inversely associated with nonelective hospitalizations but not elective hospitalization in older men in Australia. These findings point to the existence of socioeconomic inequalities in health care use, indicative of a need to take action to reduce these inequalities.

## Key Points

**Question:**

Is socioeconomic position (SEP) associated with all-cause and cause-specific elective and nonelective hospitalizations among older men?

**Findings:**

In this cohort study of 1566 men in Australia aged 70 years or older, low SEP as assessed by 3 individual SEP indicators and a cumulative score was associated with more all-cause nonelective hospitalizations and longer cumulative time in hospital for nonelective hospitalizations. No associations were found between SEP and elective hospitalizations, even though disadvantaged older people are likely to have a higher level of need.

**Meaning:**

These findings point to the existence of socioeconomic inequalities in health care use, despite the Australian universal coverage public health care system.

## Teaser

This cohort study examines the associations between socioeconomic position and elective and nonelective hospitalization among older men in Australia.

## Introduction

The rapid growth of aging populations globally has raised concerns about the health and health care needs of older adults.^1^ Hospitalization rates, the number of hospitalizations, and hospital expenditure increase with age.^2^ Equity of access to health care should be within reach of all to achieve best health in older people.^3^

The Australian public health care system is universally accessible under Medicare, which is funded by the Australian government through taxation revenue and offers fee-free treatment for public patients in a public hospital.^4^ Private health insurance is not compulsory in Australia, and approximately half of the older population has private insurance. Insurance fully or partly covers the costs of being admitted to a private hospital and/or the costs of other ancillary health services that are not subsidized by Medicare.^4^

Although the Australian public health care system has a high overall performance, it is not free of inequities in access to health services. In Australia, although public hospitals account for most nonelective (ie, unplanned) admissions, a large proportion of admissions to private hospitals are elective, which is mainly funded by private health insurance. Waiting times for elective surgery are usually shorter in a private hospital than in a public hospital, where the median waiting time was 39 days in 2019 to 2020.^5^ In other words, those with private insurance can generally afford to use private hospitals for more timely treatment for elective surgery. Inequalities in access to health services could also be due to gaps in services covered and out-of-pocket costs for services that are not covered by Medicare, or when the fee charged by service providers is higher than the rebate paid by Medicare.^6,7^ Socioeconomic position (SEP) has been proposed as an indicator of inequities in access to health services.^8^

Among older adults, in few studies, low SEP has been shown to be associated with a higher rate and risk of any hospitalization (elective and nonelective).^9,10,11,12^ Moreover, there may be differences in the influence of SEP on elective compared to nonelective hospitalizations. Low SEP was associated with a higher risk of nonelective hospitalizations among adults and older adults in Scotland^13^ and Ireland,^14^ but another study reported no association between social class and elective hospitalizations among adults in the UK aged 45 to 65 years.^15^

Given the increased rate of hospitalization among older Australians in the past decade (3.3% per year), high hospitalization expenditure among Australian men,^16^ and the limited evidence about inequitable access to hospital care based on SEP, we aimed to examine the associations of education, occupation, income, and cumulative SEP with all-cause and cause-specific elective and nonelective hospitalization among older men in Australia who lived within a defined metropolitan geographical area. We also examined the association between SEP indicators and the number of elective and nonelective hospitalizations and hospital length of stay (LOS).

## Methods

### Study Population

The Concord Health and Aging in Men Project (CHAMP) is a population-based cohort study among 1705 men aged 70 years or older living in the Local Government Areas of Burwood, Canada Bay, and Strathfield in Sydney, Australia (participation rate of 54%).^17^ CHAMP began with baseline data collection in 2005 to 2007, with 4 subsequent follow-up assessments. The only baseline exclusion criterion was living in an aged care facility. The CHAMP study complied with the World Medical Association Declaration of Helsinki^18^ and was approved by the Sydney South West Area Health Service Human Research Ethics Committee. Written informed consent was obtained from all participants involved in the study. This study followed the Strengthening the Reporting of Observational Studies in Epidemiology (STROBE) reporting guideline.

### Assessment of Socioeconomic Indicators at Baseline

We used 3 self-reported SEP indicators from early adult life to older age (education, occupation, and source of income). Education was assessed based on the highest qualification attained and classified as high (university degree), intermediate (trade, apprenticeship, certificate, or diploma), and low (no postschool qualification). The longest occupation performed during working life and the Australian and New Zealand Standard Classification of Occupations were used to create 3 main categories: high (higher professionals and managers, lower professionals and managers, and higher clerical service), intermediate (small employers and self-employed, farmers, lower supervisors, and technicians), and low (lower clerical, service, sales workers, and skilled and unskilled workers).^19^ Australia’s retirement income system comprises 3 pillars: a means-tested age pension, mandatory occupational superannuation, and voluntary long-term savings. Participants reported their sources of income by selecting from the following 6 response options (more than 1 response allowed): age pension; repatriation pension or veteran’s pension; superannuation or other private income; own business farm, or partnership; wage or salary; and other (please specify). Subsequently, sources of income were classified as high (sources of income do not include any government pension), intermediate (reliance on government pensions and other sources of income), and low (reliant solely on a government pension).

To assess the cumulative consequences of being exposed to low SEP across the life course, a cumulative SEP score was computed by summing the 3 abovementioned SEP indicators coded as 0 (high), 1 (intermediate), and 2 (low). The cumulative SEP score ranged between 0 and 6 and was divided into tertiles: high (score 0-2), intermediate (score 3-4), and low (score 5-6); a higher score indicates a greater disadvantaged SEP.

### Outcome Ascertainment

Consenting participants (n = 1639, 96%) were successfully followed up for any hospital admission using the New South Wales Admitted Patient Data Collection for all public and private hospitals in New South Wales, Australia, from January 1, 2005, to December 31, 2017.^20^ Records were linked to CHAMP baseline data by the Centre for Health Record Linkage ^21^ using probabilistic record linkage methodology and ChoiceMaker software (ChoiceMaker LLC). Death data were also obtained by the Centre for Health Record Linkage from the New South Wales Registry of Births, Deaths, and Marriages.

The primary outcomes were any first elective and nonelective hospitalizations following recruitment to CHAMP, defined as at least an overnight hospital stay; a given participant could be included for both elective and nonelective analysis. Elective and nonelective hospitalizations were differentiated by coding of urgency of admission. Cause-specific first elective and nonelective hospitalizations were coded according to the *International Classification of Diseases and Related Health Problems, Tenth Revision, Australian Modification* (*ICD-10-AM*).^22^ We focused on 14 major categories and chapters based on the organ system involved: infectious, neoplasm, hematologic, endocrine, psychiatric, neurologic, circulatory, respiratory, digestive, skin-related diseases, musculoskeletal, genitourinary, injury, and abnormal symptoms (signs, abnormal results of clinical or other research procedures, and ill-defined conditions that are not classified elsewhere)^23^ (eTable 1 in the Supplement). We report associations for the top 5 most frequent causes. Numbers of recorded elective and nonelective hospitalizations were also examined. The length of stay (the number of days between hospital admission and hospital discharge) of first all-cause elective and nonelective and the cumulative LOS of all elective and nonelective hospitalizations during follow-up were secondary outcomes.

### Assessment of Covariates

We included age, country of birth (Australian-born or other), and marital status (single; married or de facto; and widowed, separated, or divorced) as potential confounders. A priori, we decided not to adjust for most established hospitalization risk factors, including frailty and chronic conditions, as these factors are likely to be intermediate variables of the association between SEP and hospitalizations.^24^

### Statistical Analysis

Statistical analyses were performed using R, version 1.3.1056 (R Foundation for Statistical Computing); R packages include cmprsk,^25^ survival,^26^ and MASS.^27^ The associations between SEP and participants having at least 1 all-cause and cause-specific elective and nonelective hospitalizations were examined in separate models using Fine and Gray competing-risks survival regression.^28^ Adjusted subhazard ratios (SHRs) and 95% CIs are reported. Time at risk was calculated from date of baseline data collection to death, date of official withdrawal from the study, or December 31, 2017. All Fine and Gray competing-risks survival regression models were adjusted for age, age squared (to consider accelerated hospitalization rate by age), country of birth, and marital status. The proportional subhazards assumption was assessed using Schoenfeld residuals, and in all models this assumption was satisfied.^29^ Linear trends were assessed by using orthogonal polynomial contrasts. Rate ratios (RRs) for number of hospitalizations (elective and nonelective) were estimated using negative binomial regression analysis. We also used negative binomial regression to estimate the RRs and 95% CIs of the association of SEP with first elective and nonelective hospital LOS and cumulative LOS (total count of overnight hospital stays during follow-up); zero values were included for those who had no hospitalization. Negative binomial regression considers the skewness of the outcome and allows for overdispersion. For numbers of hospitalizations and cumulative LOS, we used a logarithm function of follow-up duration as the offset variable. We examined the interaction between SEP and country of birth for all analyses using the Wald test. Statistical significance was considered for a 2-sided test with a *P* < .05 for interaction.

## Results

The study sample included 1566 participants (mean [SD] age, 76.8 [5.4] years); the demographic characteristics of participants by SEP are shown in the Table. Of 1705 participants at baseline, we excluded 139 participants who did not agree to data linkage and those who had missing data for SEP or covariates (eFigure 1 in the Supplement). Excluded participants tended to be older, born outside Australia, and have lower levels of education, occupation, and sources of income compared with included participants (eTable 2 in the Supplement).

**Table.** Characteristics of Participants by Indicators of Socioeconomic Position at Baseline

Characteristic	Total population (N = 1566)	Participants, No. (%)											
		Education^a^			Occupation^b^			Source of income^c^			Tertile groups of cumulative SEP^d^		
		High (n = 190)	Intermediate (n = 672)	Low (n = 704)	High (n = 475)	Intermediate (n = 589)	Low (n = 502)	High (n = 695)	Intermediate (n = 262)	Low (n = 609)	High (n = 520)	Intermediate (n = 626)	Low (n = 420)
Age, mean (SD), y	76.8 (5.4)	76.5 (4.9)	76.7 (5.5)	76.9 (5.4)	76.9 (5.4)	76.7 (5.5)	76.8 (5.1)	76.9 (5.6)	76.6 (5.2)	76.7 (5.2)	76.7 (5.3)	77.1 (5.7)	76.5 (4.9)
Age group													
70-79	1130	136 (71.6)	492 (73.2)	502 (71.3)	338 (71.2)	434 (73.7)	358 (71.3)	475 (68.3)	196 (74.8)	459 (75.4)	374 (71.9)	433 (69.2)	323 (76.9)
≥80	436	54 (28.4)	180 (26.8)	202 (28.7)	137 (28.8)	155 (26.3)	144 (28.7)	220 (31.7)	66 (25.2)	150 (24.6)	146 (28.1)	193 (30.8)	97 (23.1)
Country of birth													
Australia	794	125 (65.8)	396 (58.9)	273 (38.8)	325 (68.4)	270 (45.8)	199 (39.6)	439 (63.2)	167 (63.7)	188 (30.9)	355 (68.3)	330 (52.7)	109 (26.0)
Other^e^	772	65 (34.2)	276 (41.1)	431 (61.2)	150 (31.6)	319 (54.2)	303 (60.4)	256 (36.8)	95 (36.3)	421 (69.1)	165 (31.7)	296 (47.3)	311 (74.0)
Marital status													
Single	82	14 (7.4)	32 (4.8)	36 (5.1)	26 (5.5)	33 (5.6)	23 (4.6)	35 (5.0)	16 (6.1)	31 (5.1)	26 (5.0)	42 (6.7)	14 (3.3)
Married or de facto	1207	154 (81.1)	509 (75.7)	544 (77.3)	378 (79.6)	430 (73.0)	399 (79.5)	549 (79.0)	199 (76.0)	459 (75.4)	418 (80.4)	457 (73.0)	332 (79.0)
Widowed separated, or divorced	277	22 (11.6)	131 (19.5)	124 (17.6)	71 (14.9)	126 (21.4)	80 (15.9)	111 (16.0)	47 (17.9)	119 (19.5)	76 (14.6)	127 (20.3)	74 (17.6)

Abbreviation: SEP, socioeconomic position.

^a^ Education categorized as high (university degree), intermediate (trade, apprenticeship, certificate or diploma), and low (no postschool qualification).

^b^ Occupation categorized as high (higher professionals and managers, lower professionals and managers, and higher clerical service), intermediate (small employers and self-employed, farmers, lower supervisors, and technicians), and low (lower clerical, service, sales workers, skilled and unskilled workers).

^c^ Source of income categorized as high (sources of income do not include any government pension), intermediate (reliance on government pensions and other sources of income), and low (reliant solely on a government pension).

^d^ Tertile groups of cumulative SEP categorized as high (cumulative SEP score 0-2), intermediate (cumulative SEP score 3-4), and low (cumulative SEP score 5-6).

^e^ Predominantly includes migrants born in China, Great Britain, Italy, and Greece.

### Elective Hospitalization

#### Association of SEP With All-Cause and Cause-Specific Elective Hospitalization

**Figure 1.**
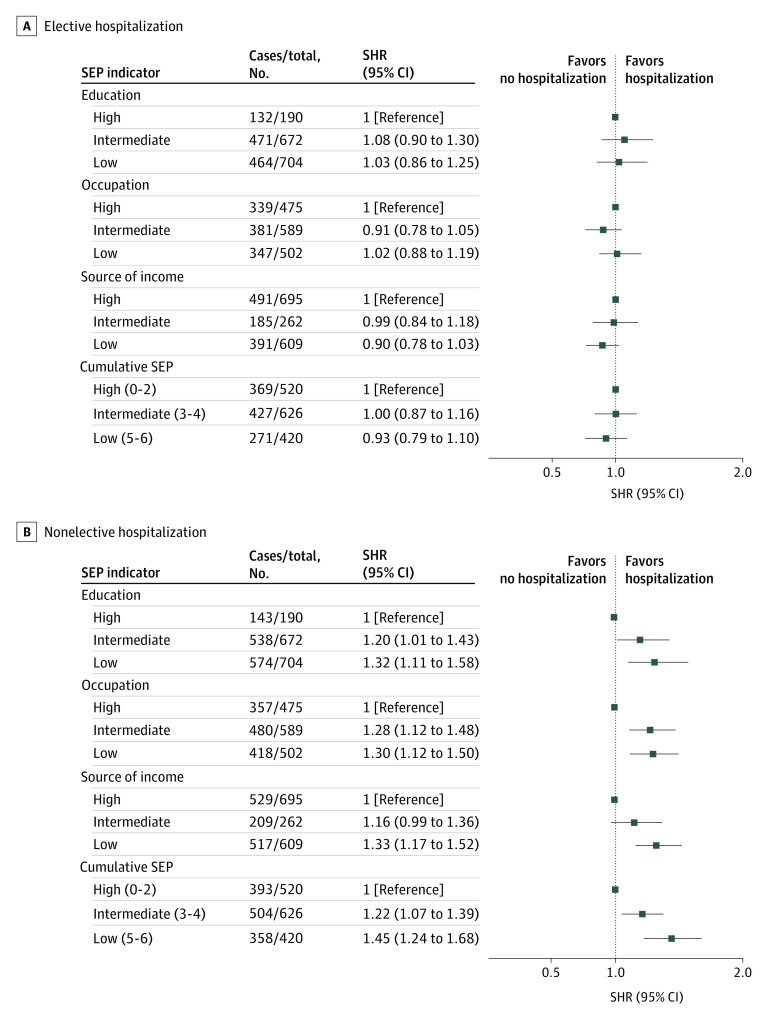
Association of Socioeconomic Indicators With Having at Least 1 Elective or Nonelective Hospitalization Among 1566 Participants Calendar year was used as the time scale, with survivors having a censoring date of December 31, 2017, for elective hospitalization (8189 person-years of follow-up) and nonelective hospitalization (8282 person-years of follow-up). All estimates were adjusted for age, age squared, country of birth, and marital status. SEP indicates socioeconomic position; SHR, subhazard ratio.

During a mean (SD) 9.07 (3.53) years of follow-up, 1067 of 1566 participants had at least 1 elective hospitalization (eTable 3 in the Supplement), with the median first elective LOS of 3.0 days (IQR, 1.0-8.0 days). There was no statistically significant association between SEP indicators and having at least 1 elective hospitalization (Figure 1). Out of 14 major categories of organ system, neoplasm, circulatory, digestive, musculoskeletal, and genitourinary were the most frequent causes for first elective hospitalization (eFigure 2 in the Supplement). No statistically significant associations were evident between SEP indicators and cause-specific elective hospitalization (eFigure 3 in the Supplement).

#### Association of SEP With Number of Elective Hospitalizations

**Figure 2.**
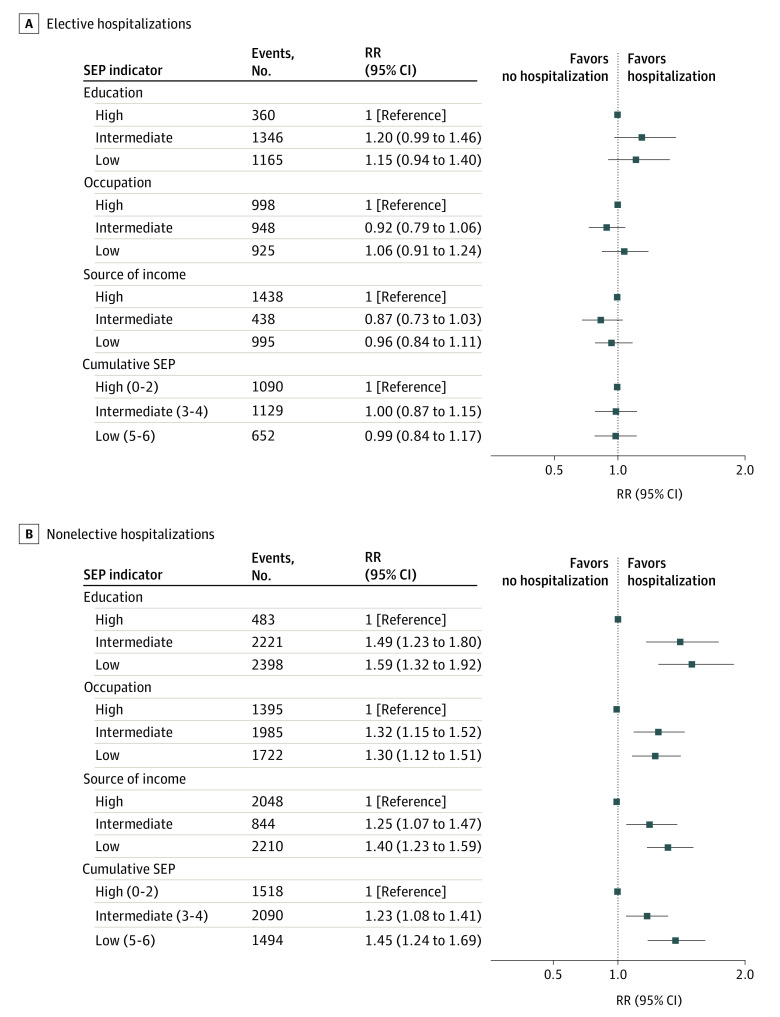
Association of Socioeconomic Indicators With Number of Elective and Nonelective Hospitalizations Overall, the total number of elective hospitalizations was 2871 and nonelective hospitalization was 5102. All estimates were adjusted for age, age squared, country of birth, and marital status. RR indicates rate ratio; SEP, socioeconomic position.

Overall, there were 2871 records of elective hospitalizations during follow-up, with the median cumulative LOS of 10.0 days (IQR, 3.0-24.0 days). No association was evident between SEP indicators and the number of elective hospitalizations (Figure 2).

#### Association of SEP With LOS of Elective Hospitalizations

**Figure 3.**
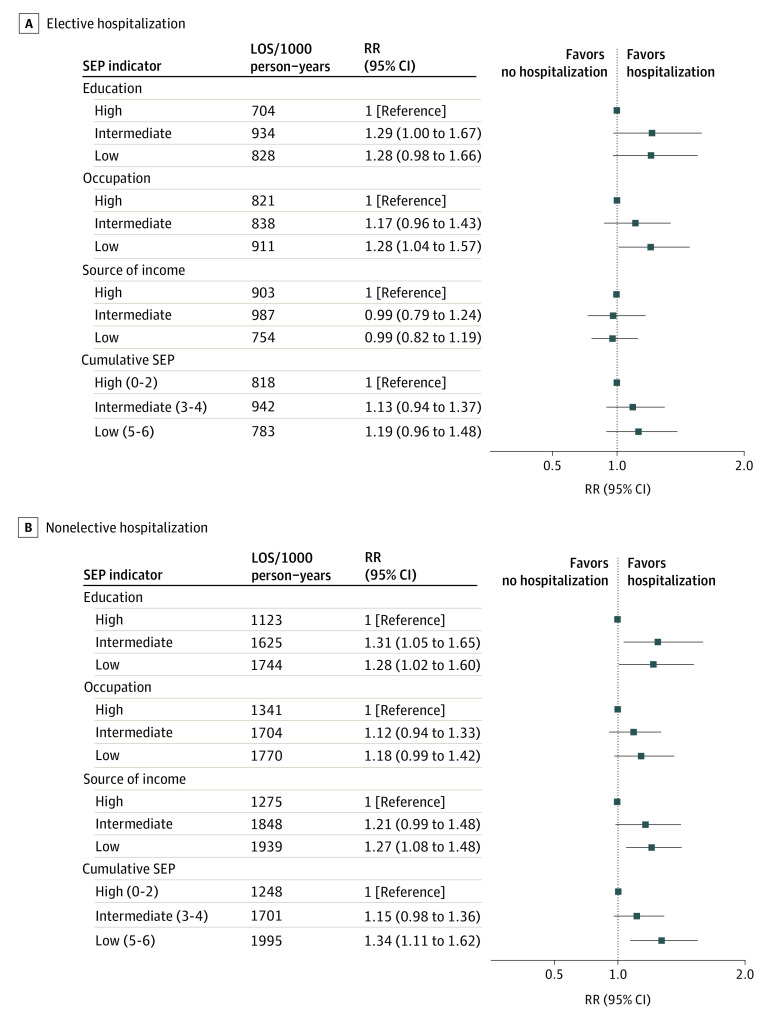
Association of Socioeconomic Indicators With Length of Stay of First Elective and Nonelective Hospitalization All estimates were adjusted for age, age squared, country of birth, and marital status. LOS indicates length of stay; RR, rate ratio; and SEP, socioeconomic position.

**Figure 4.**
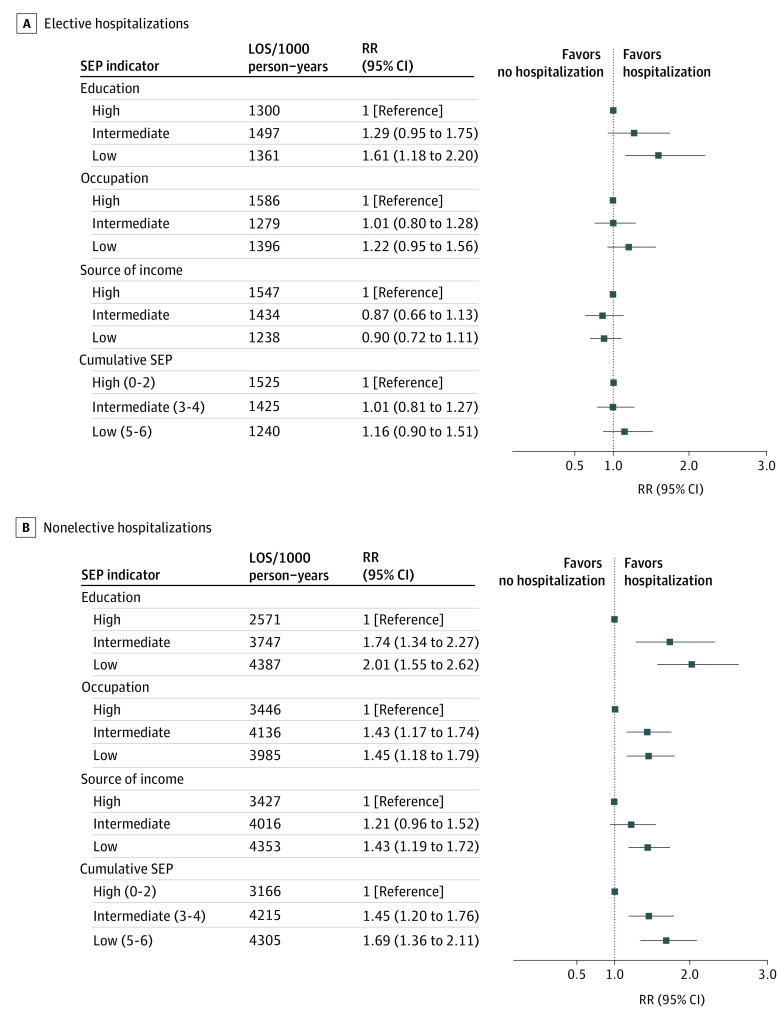
Association of Socioeconomic Indicators With Cumulative Length of Stay of Elective and Nonelective Hospitalizations All estimates were adjusted for age, age squared, country of birth, and marital status. LOS indicates length of stay; RR, rate ratio; and SEP, socioeconomic position.

No associations were evident between SEP indicators and LOS of first elective hospitalization, except for education (compared with the high educational level, relative risks [RRs], 1.29; 95% CI, 1.00-1.67 for intermediate and 1.28; 95% CI, 0.98-1.66 for low) and occupational position (compared with the high occupation level, RRs, 1.17; 95% CI, 0.96-1.43 for intermediate and 1.28; 95% CI, 1.04-1.57 for low) (Figure 3). For cumulative LOS of elective hospitalizations, the only association of an SEP indicator was for education (compared with the high education level, RRs, 1.29; 95% CI, 0.95-1.75 for intermediate and 1.61; 95% CI, 1.18-2.20 for low) (Figure 4).

### Nonelective Hospitalization

#### Association of SEP With All-Cause and Cause-Specific Nonelective Hospitalization

During follow-up, 1255 of 1566 participants had at least 1 nonelective hospitalization (eTable 3 in the Supplement), with a median first nonelective LOS of 5.0 days (IQR, 3.0-11.0 days). There was an inverse association across SEP groups as assessed by educational level, occupational position, sources of income, and cumulative SEP and having at least 1 nonelective hospitalization (*P* < .001 for trend) (Figure 1). Out of 14 major categories of organ system, circulatory, injury, abnormal symptoms, respiratory, and digestive were the most frequent causes for first nonelective hospitalization (eFigure 2 in the Supplement). The only statistically significant associations were between being in the intermediate (SHR, 1.92; 95% CI, 1.20-3.08) or low (SHR, 1.96; 95% CI, 1.31-2.93) source of income groups and having higher risk of nonelective hospitalization because of respiratory conditions (eFigure 4 in the Supplement). There was a tendency toward a higher risk of nonelective hospitalization due to circulatory conditions among those with low educational level and occupational position, although this finding was not statistically significant.

#### Association of SEP With Number of Nonelective Hospitalizations

Overall, there were 5102 records of nonelective hospitalizations during follow-up, with the median cumulative LOS of 26.0 days (IQR, 9.0-60.5 days). There were statistically significant associations between being in the lowest educational level (RR, 1.59; 95% CI, 1.32-1.92 vs high educational level), occupational position (RR, 1.30; 95% CI, 1.12-1.51 vs high occupation level), sources of income (RR, 1.40; 95% CI, 1.23-1.59 vs high source of income level), and cumulative SEP (RR, 1.45; 95% CI, 1.24-1.69 vs high cumulative SEP) and having an increased number of nonelective hospitalizations (Figure 2).

#### Association of SEP With LOS of Nonelective Hospitalizations

Significant associations were evident between being in the lowest SEP groups and having longer LOS of first nonelective hospitalization (RRs, 1.28; 95% CI, 1.02-1.60 for lowest vs the highest educational level; 1.18; 95% CI, 0.99-1.42 for the lowest vs the highest occupation; 1.27; 95% CI, 1.08-1.48 for the lowest vs the highest income; and 1.34; 95% CI, 1.11-1.62 for the lowest vs the highest cumulative SEP tertile) (Figure 3). Moreover, being in low SEP groups was associated with a higher risk of having longer cumulative LOS of nonelective hospitalizations (RRs, 2.01; 95% CI, 1.55-2.62 for lowest vs the highest educational level; 1.45; 95% CI, 1.18-1.79 for the lowest vs the highest occupation; 1.43; 95% CI, 1.19-1.72 for the lowest vs the highest income; and 1.69; 95% CI, 1.36-2.11 for the lowest vs the highest cumulative SEP tertile) (Figure 4).

## Discussion

In this cohort study, we found that being in low SEP groups was associated with at least 1 nonelective hospitalization, number of nonelective hospitalizations, and cumulative LOS for nonelective hospitalizations. Of all the most common cause-specific hospitalizations, the only statistically significant finding was an inverse association between source of income and nonelective hospitalization due to respiratory conditions. We found no association of SEP indicators with having at least 1 elective hospitalization, cause-specific elective hospitalization, number of elective hospitalizations, and LOS of elective hospitalizations.

### Current Findings in Context of Other Evidence

#### Association of SEP With All-Cause and Cause-Specific Elective and Nonelective Hospitalization

We found that having at least 1 elective hospitalization was not socially patterned, which agrees with the only published prospective study among UK adults (aged 45 to 65 years) evaluating the association between social class and planned hospitalization.^15^ We also found no association between SEP indicators and number of elective hospitalizations. Given the greater prevalence of some reasons for elective hospitalization (eg, cardiac procedures, joint replacement) and of risk factors for hospitalizations among those in low SEP groups (eg, comorbidities), these results suggest that access to elective hospitalizations is not in proportion to need—with the least advantaged groups being underserved and the most advantaged groups overusing services.^3^ To our knowledge, no other studies have focused on socioeconomic inequalities in cause-specific elective hospitalization.

We found associations between low SEP and nonelective hospitalization. Our results are consistent with previous studies showing that socioeconomic deprivation is inversely associated with unplanned or emergency admissions among adults and older adults.^13,14^ We found that cumulative exposure to socioeconomic disadvantage over the life course was associated with nonelective hospitalization, mainly driven by the effect of education and income; to our knowledge, there have been no previous studies of this association.

Considering each SEP indicator, limited evidence exists for nonelective hospitalizations; our study adds to this body of evidence by examining and comparing patterns for both elective and nonelective hospitalizations. Similar to the previously published studies on any hospitalizations,^30,31,32^ we found a significant association between having a low educational level and higher risk of nonelective hospitalization. To date, there is no study examining the association between occupational position and nonelective hospitalization among older adults. However, the higher risk of nonelective hospitalization among those with low occupational position in our study is consistent with previous studies on any hospitalizations among young adults in Spain^33^ and Finland.^34^ In addition, our study found higher risk of nonelective hospitalizations for men reliant on a government pension solely (a relatively low level of income), which is in line with a retrospective cohort study among patients 65 years or older in the Netherlands^11^ and a study among 339 US adults with a mean age of 72.3 years focusing on any hospitalization.^35^

Regarding cause-specific nonelective hospitalization, we found an association between sources of income and nonelective hospitalization due to respiratory conditions, which is in agreement with previous studies on any cause-specific hospitalizations in Europe.^10,36^ We also found a tendency toward a higher risk of nonelective hospitalization because of respiratory diseases among those with a low educational level which agrees with results from other studies on any hospitalizations.^36,37^ These associations could be explained potentially in part by higher smoking rates among lower socioeconomic groups.^38^

Our results showed a tendency (not statistically significant) toward higher risk of nonelective hospitalization due to circulatory conditions among those with low educational level and occupational position, which agrees with previous studies in Europe, UK, and the US.^10,15,39,40^ This finding is consistent with the well-established association between low SEP and increased risk of cardiovascular disease.^41^

#### Association of SEP With LOS

Research on the association between SEP and longer LOS for nonelective hospitalizations among community-dwelling older adults is limited. Our study shows that having a low educational level and occupational position, having a limited source of income, and being in the lowest tertile of cumulative SEP were significantly associated with a longer cumulative LOS for nonelective hospitalizations (ie, spending more time in hospital). These results are consistent with previous studies among older adults in Canada,^9^ Rome,^10^ and the Netherlands.^11^

### Possible Mechanisms

Overall, the inverse association between SEP and risk of hospitalization has several potential explanations. Socioeconomically disadvantaged individuals are more likely to be exposed to unhealthy diets, tobacco consumption, and low physical activity levels and therefore accumulate risk factors of morbidity^42,43^ that are of greater complexity and severity.^15,44,45^ Moreover, higher levels of nonelective hospitalization among those with low SEP may also be related to lack of health literacy, health care seeking behavior, and having low levels of social support.^46,47,48^ Previous CHAMP studies have shown that health-related behaviors, multimorbidity, and social support are patterned by socioeconomic indicators.^48,49,50^ Moreover, more people with a low SEP than a high SEP lack private health insurance. This is an important issue, as having private health insurance enhances access to timely elective health care, which might reduce the number of nonelective hospitalizations.^51^ Persons without private health insurance would have to pay the complete cost of private hospital admissions (other than the Medicare rebate for a clinician’s services) or wait to have their elective hospitalization in a public hospital. High out-of-pocket cost of health care for privately performed elective procedures in Australia could be another explanation.^52^ Increasing copayments lead to people forgoing medical care, mainly among the least advantaged groups.^53^ Further studies are needed to examine the mediating role of multimorbidity, poor health-related behaviors, and health insurance status on SEP inequalities in hospitalizations.

Indicators of SEP can influence hospitalizations differently. Occupational status could have an important effect on the health of older adults.^45^ Low occupation level might cause physical (eg, osteoarthritis) and psychosocial risks and subsequently lead to a higher risk of hospitalization (eg, elective joint replacement surgery).^54^ Finally, income directly affects the availability of health resources that can reduce the risk and number of hospitalizations.^55^ Moreover, forgoing planned health care owing to financial reasons (ie, out-of-pocket payments) is highly prevalent worldwide and in Australia.^56^

### Strengths and Limitations

To our knowledge, this is the first study evaluating the association of 3 individual SEP indicators as well as a cumulative SEP score from early adult life to older age with all-cause and cause-specific elective and nonelective hospitalization. The study had the benefit of a long follow-up period and high-quality record linkage. Moreover, men recruited to CHAMP are similar to the general population of older men in Australia.^17^ We considered death as a competing risk factor for any and cause-specific elective and nonelective hospitalization.

This study also has some limitations. First, CHAMP only included men 70 years or older, so the results may not be generalizable to women or people younger than 70 years. These results may also not be generalizable to rural and regional populations. Second, although we included 3 individual indicators of SEP and 1 cumulative SEP score, we lack data on wealth, which is a better SEP indicator at older age to capture variations in financial security.^57^ Moreover, we do not have data on working conditions, such as job stability, working hours, and shift work, which may more comprehensively reflect the occupational position of an individual.^49^ We also do not have data on private insurance; having private insurance will allow people, regardless of SEP, to enhance access to special health services and timely access to health care.^51^ Third, hospitalizations were defined as at least 1 overnight hospital stay; thus, the results may not be generalizable to day-only admissions. Fourth, owing to the small number of cause-specific hospitalization, the power of our study was low for some outcomes.

## Conclusions

In this cohort study, low SEP as assessed by 3 individual SEP indicators and a cumulative score was associated with more all-cause nonelective hospitalizations and longer cumulative time in hospital for nonelective hospitalizations among older men in Australia. No associations were observed for elective hospitalization, even though disadvantaged older people are likely to have a higher level of need. These findings point to the existence of socioeconomic inequalities in health care use, indicative of a need to take action to reduce these inequalities.

## Notes

Supplement. **eTable 1.** Included ICD-10 major categories based on organ system involved **eTable 2.** Characteristics of participants included and excluded from the analysis, CHAMP **eTable 3.** Baseline characteristics of participants by having at least 1 elective and non-elective hospitalization, CHAMP **eFigure 1.** Sample selection flow chart **eFigure 2.** Distribution of the top five cause-specific first elective and non-elective hospitalization **eFigure 3.** Association of socioeconomic position indicators with first cause-specific elective hospitalization, CHAMP **eFigure 4.** Association of socioeconomic position indicators with first cause-specific non-elective hospitalization, CHAMP
