# Supplementary material for: Socioeconomic Inequalities in Elective and Nonelective Hospitalizations in Older Men
Source: JAMA Netw Open. 2022 Apr 7;5(4):e226398. doi: 10.1001/jamanetworkopen.2022.6398 (PMC8990350; doi:10.1001/jamanetworkopen.2022.6398)
Supplement: Supplement. — eTable 1. Included ICD-10 major categories based on organ system involved eTable 2. Characteristics of participants included and excluded from the analysis, CHAMP eTable 3. Baseline characteristics of participants by having at least 1 elective and non-elective hospitalization, CHAMP eFigure 1. Sample selection flow chart eFigure 2. Distribution of the top five cause-specific first elective and non-elective hospitalization eFigure 3. Association of socioeconomic position indicators with first cause-specific elective hospitalization, CHAMP eFigure 4. Association of socioeconomic position indicators with first cause-specific non-elective hospitalization, CHAMP [file jamanetwopen-e226398-s001.pdf]

Supplemental Online Content

Xu P, Blyth FM, Naganathan V, et al. Socioeconomic inequalities in elective and nonelective hospitalizations in older men. *JAMA Netw Open*. 2022;5(4):e226398.
doi:10.1001/jamanetworkopen.2022.6398

eTable 1. Included *ICD-10* Major Categories Based on Organ System Involved

eTable 2. Characteristics of Participants Included and Excluded From the Analysis, CHAMP

eTable 3. Baseline Characteristics of Participants by Having at Least 1 Elective and Nonelective Hospitalization, CHAMP

eFigure 1. Sample Selection Flowchart

eFigure 2. Distribution of the Top 5 Cause-Specific First Elective and Nonelective Hospitalization

eFigure 3. Association of Socioeconomic Position Indicators With First Cause-Specific Elective Hospitalization, CHAMP

eFigure 4. Association of Socioeconomic Position Indicators With First Cause-Specific Nonelective Hospitalization, CHAMP

This supplemental material has been provided by the authors to give readers additional information about their work.

© 2022 Xu P et al. *JAMA Network Open.*

eTable 1. Included ICD-10 major categories based on organ system involved.

Major categories/chapters	ICD-10 code
Infectious	A00.00 - A99.99, B00.00 - B99.99
Neoplasm	C00.00 - C97.99, D00.00 - D48.99
Hematologic	D50.00 - D89.99
Endocrine	E00.00 - E90.99
Psychiatric	F00.00 - F99.99
Neurologic	G00.00 - G99.99
Circulatory	I00.00 - I99.99
Respiratory	J00.00 - J99.99
Digestive	K00.00 - K93.99
Skin-related diseases	L00.00 - L99.99
Musculoskeletal	M00.00 - M99.99
Genitourinary	N00.00 - N99.99
Injury	S00.00 - S99.99, T00.00 - T98.99
Abnormal symptoms*	R00.00 - R99.99

*Abnormal symptoms define as symptoms, signs, abnormal results of clinical or other research procedures, and ill-defined conditions that are not classified elsewhere.

© 2022 Xu P et al. *JAMA Network Open.*

eTable 2. Characteristics of participants included and excluded from the analysis, CHAMP.

Characteristic	Included, %	Excluded, %	P-value[a]
	(n=1566)	(n=139)	
Age, *years*	76.8 ± 5.4	76.3 ± 5.2	0.29
Age group			0.001
70-79	72.2	59.0	
80+	27.8	41.0	
Country of birth			0.015
Australian-born	50.7	39.6	
Other	49.3	60.4	
Living condition[b]			0.24
Live with others	81.6	69.1	
Live alone	18.4	20.9	
Marital status			0.12
Single	5.2	2.9	
Married/defacto	77.1	73.4	
Widowed/separated/divorced	17.7	23.7	
Education[b,c]			0.002
High	12.1	11.5	
Intermediate	42.9	23.7	
Low	45.0	52.5	
Occupation[b,d]			0.003
High	30.3	21.6	
Intermediate	37.6	27.3	
Low	32.1	43.2	
Source of income[b,e]			0.004
High	44.4	33.8	
Intermediate	16.7	5.8	
Low	38.9	42.5	
Tertile groups of Cumulative SEP[f]			0.57
High	33.2	23.0	
Intermediate	40.0	22.3	
Low	26.8	19.4	

Abbreviations: CHAMP, Concord Health and Ageing in Men Project; SEP, socioeconomic position.

Data are mean ± SD for continuous variables or percent for categorical variables.

[a] P-value calculated using Chi-square test for categorical variables and student's t-test for continuous variables.

[b] Due to some missing data, percentages do not always add to 100%.

cEducation categorized as 'high' (university degree), 'intermediate' (trade, apprenticeship, certificate or diploma) and 'low' (no post-school qualification).

[d] Occupation categorized as 'high' (higher professionals and managers, lower professionals and managers, and higher clerical service), 'intermediate' (small employers and self-employed, farmers, lower supervisors and technicians), and 'low' (lower clerical, service, sales workers, skilled and unskilled workers).

[e] Source of income categorized as 'high' (sources of income do not include any government

© 2022 Xu P et al. *JAMA Network Open.*

pension), ‘intermediate’ (reliance on government pensions and other sources of income) and ‘low’ (reliant solely on a government pension).

[f] Tertile groups of Cumulative SEP categorized as ‘high’ (cumulative SEP score 0-2), ‘intermediate’ (cumulative SEP score 3-4) and ‘low’ (cumulative SEP score 5-6).

© 2022 Xu P et al. *JAMA Network Open.*

eTable 3. Baseline characteristics of participants by having at least one elective and non-elective hospitalization, CHAMP.

Characteristics	Elective hospitalization, %			Non-elective hospitalization, %	
	No (n=499)	Yes (n=1067)		No (n=311)	Yes (n=1255)
Age, years	77.7±6.0	76.4±5.0		74.8±5.0	77.3±5.3
Age group					
70-79 (n=1130)	65.7	75.2		84.6	69.1
80+ (n=436)	34.3	24.8		15.4	30.9
Country of birth					
Australian-born (n=794)	41.3	55.1		49.2	51.1
Other (n=772)	58.7	44.9		50.8	48.9
Marital status					
Single (n=82)	5.4	5.2		7.1	4.8
Married/defacto (n=1207)	77.2	77.0		82.6	75.7
Widowed/separated/divorced (n=277)	17.4	17.8		10.3	19.5
Education					
High (n=190)	11.6	12.4		15.1	11.4
Intermediate (n=672)	40.3	44.1		43.1	42.9
Low (n=704)	48.1	43.5		41.8	45.7
Occupation					
High (n=475)	27.3	31.8		37.9	28.4
Intermediate (n=589)	41.7	35.7		35.0	38.2
Low (n=502)	31.1	32.5		27.0	33.3
Source of income					
High (n=695)	40.9	46.0		53.4	42.2
Intermediate (n=262)	15.4	17.3		17.0	16.7
Low (n=609)	43.7	36.6		29.6	41.2
Tertile groups of Cumulative SEP					
High (n=520)	30.3	34.6		40.8	31.3
Intermediate (n=626)	39.9	40.0		39.2	40.2
Low (n=420)	29.9	25.4		19.9	28.5

Abbreviations: CHAMP, Concord Health and Ageing in Men Project; SEP, socioeconomic position.

N = 1566. Data are mean ± SD for continuous variables or percent for categorical variables. Education categorized as 'high' (university degree), 'intermediate' (trade, apprenticeship, certificate or diploma) and 'low' (no post-school qualification); Occupation categorized as 'high' (higher professionals and managers, lower professionals and managers, and higher clerical service), 'intermediate' (small employers and self-employed, farmers, lower supervisors and technicians), and 'low' (lower clerical, service, sales workers, skilled and unskilled workers); Source of income categorized as 'high' (sources of income do not include any government

© 2022 Xu P et al. *JAMA Network Open.*

pension), ‘intermediate’ (reliance on government pensions and other sources of income) and ‘low’ (reliant solely on a government pension); Tertile groups of Cumulative SEP categorized as ‘high’ (cumulative SEP score 0-2), ‘intermediate’ (cumulative SEP score 3-4) and ‘low’ (cumulative SEP score 5-6).

© 2022 Xu P et al. *JAMA Network Open.*

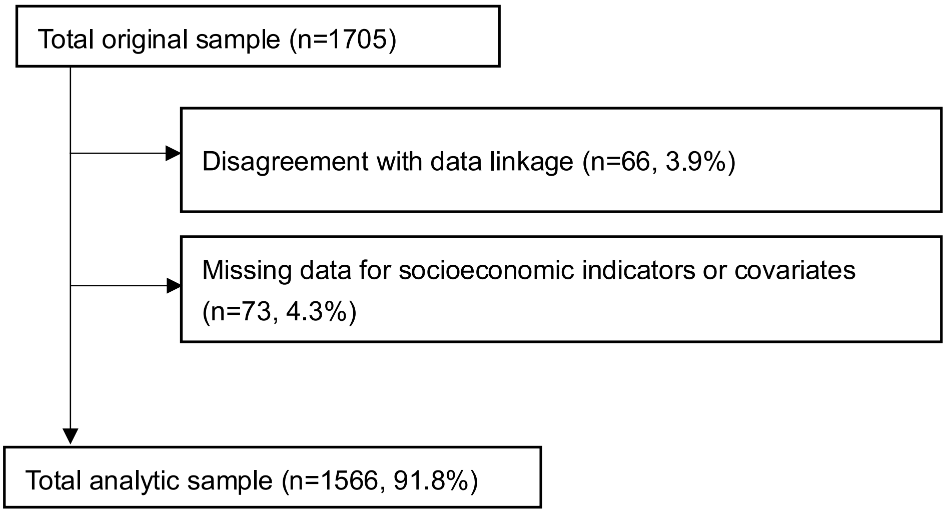

eFigure 1. Sample selection flow chart.

© 2022 Xu P et al. *JAMA Network Open.*

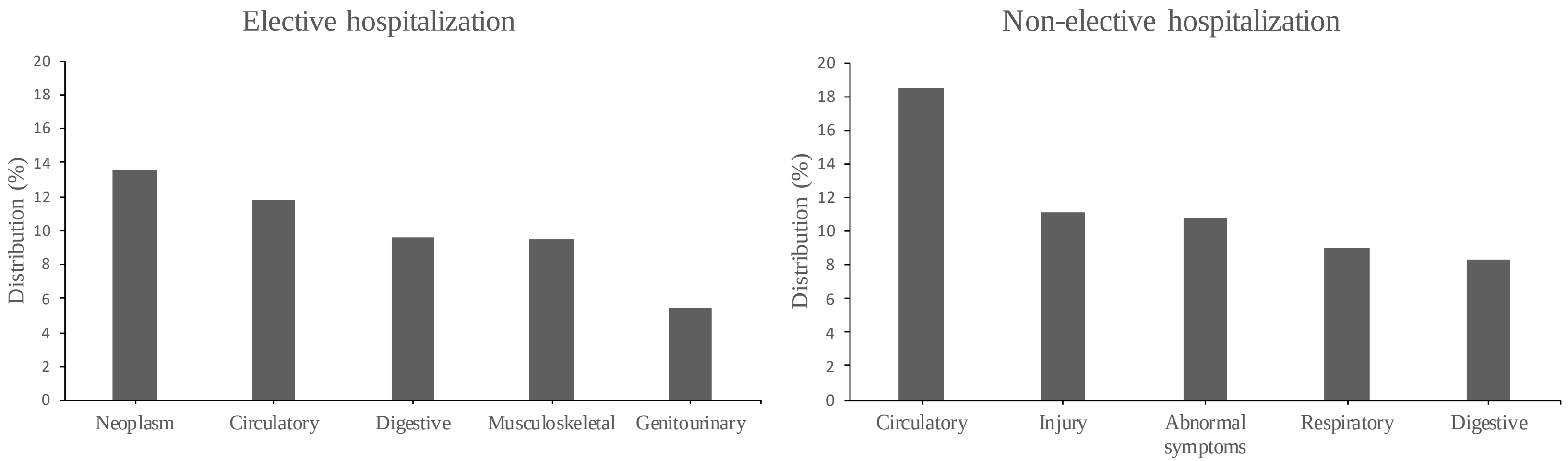

eFigure 2. Distribution of the top five cause-specific first elective and non-elective hospitalization.

Cause-specific elective and non-elective hospitalizations were classified using the International Classification of Diseases and Related Health Problems, Tenth Revision, Australian Modifications (ICD-10-AM) primary diagnosis classification: neoplasm (C00.00-C97.99, D00.00-D48.99), circulatory (I00.00-I99.99), respiratory (J00.00-J99.99), digestive (K00.00-K93.99), musculoskeletal (M00.00-M99.99), genitourinary (N00.00-N99.99), injury (S00.00-S99.99 and T00.00-T98.99), and abnormal symptoms (R00.00-R99.99; symptoms, signs, abnormal results of clinical or other research procedures, and ill-defined conditions that are not classified elsewhere).

© 2022 Xu P et al. *JAMA Network Open.*

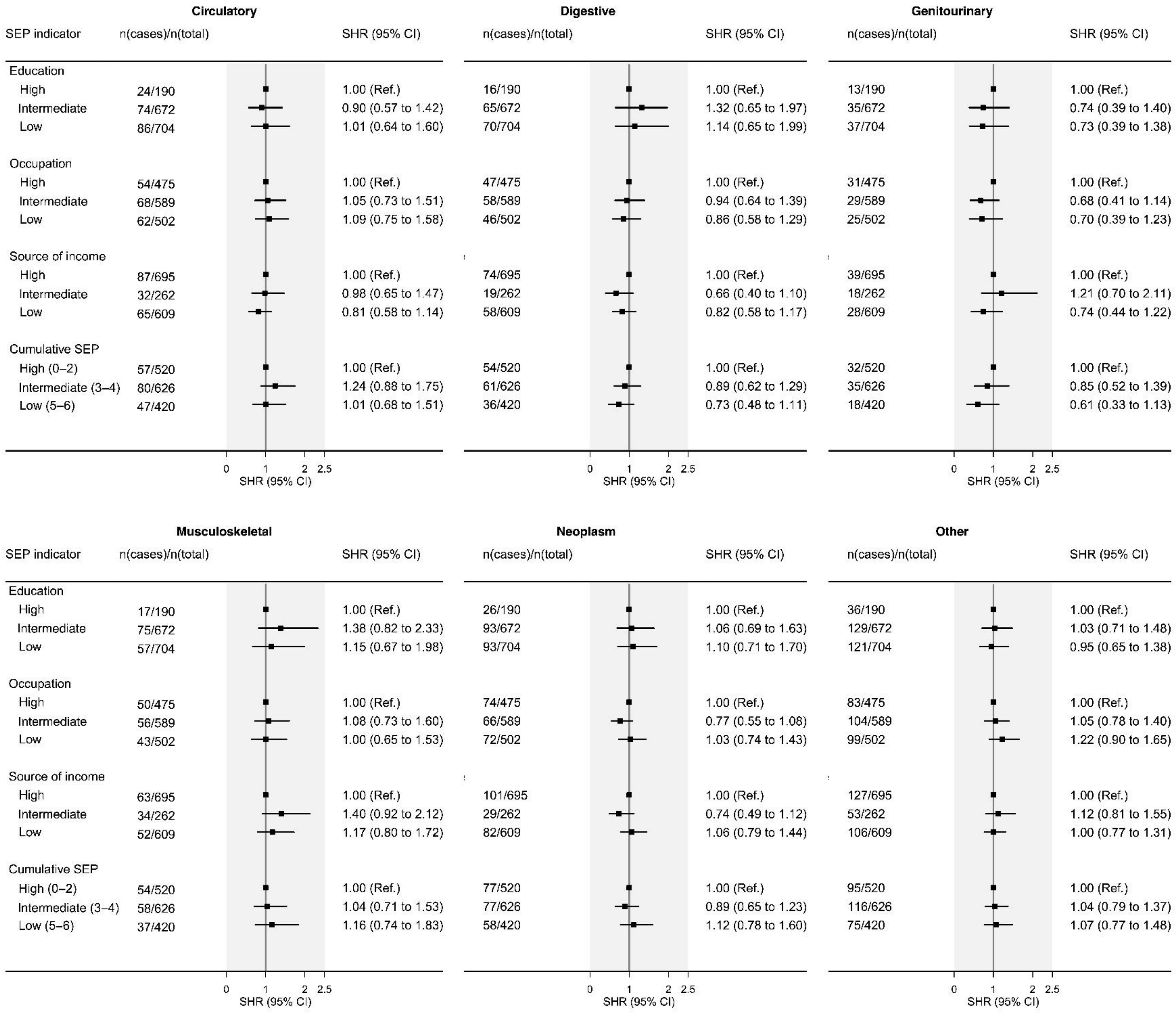
Circulatory
SEP indicator n(cases)/n(total) SHR (95% CI)
Education
High 24/190 1.00 (Ref.)
Intermediate 74/672 0.90 (0.57 to 1.42)
Low 86/704 1.01 (0.64 to 1.60)
Occupation
High 54/475 1.00 (Ref.)
Intermediate 68/589 1.05 (0.73 to 1.51)
Low 62/502 1.09 (0.75 to 1.58)
Source of income
High 87/695 1.00 (Ref.)
Intermediate 32/262 0.98 (0.65 to 1.47)
Low 65/609 0.81 (0.58 to 1.14)
Cumulative SEP
High (0–2) 57/520 1.00 (Ref.)
Intermediate (3–4) 80/626 1.24 (0.88 to 1.75)
Low (5–6) 47/420 1.01 (0.68 to 1.51)
0 1 2 2.5
SHR (95% CI)
Digestive
n(cases)/n(total) SHR (95% CI)
16/190 1.00 (Ref.)
65/672 1.32 (0.65 to 1.97)
70/704 1.14 (0.65 to 1.99)
47/475 1.00 (Ref.)
58/589 0.94 (0.64 to 1.39)
46/502 0.86 (0.58 to 1.29)
74/695 1.00 (Ref.)
19/262 0.66 (0.40 to 1.10)
58/609 0.82 (0.58 to 1.17)
54/520 1.00 (Ref.)
61/626 0.89 (0.62 to 1.29)
36/420 0.73 (0.48 to 1.11)
0 1 2 2.5
SHR (95% CI)
Genitourinary
n(cases)/n(total) SHR (95% CI)
13/190 1.00 (Ref.)
35/672 0.74 (0.39 to 1.40)
37/704 0.73 (0.39 to 1.38)
31/475 1.00 (Ref.)
29/589 0.68 (0.41 to 1.14)
25/502 0.70 (0.39 to 1.23)
39/695 1.00 (Ref.)
18/262 1.21 (0.70 to 2.11)
28/609 0.74 (0.44 to 1.22)
32/520 1.00 (Ref.)
35/626 0.85 (0.52 to 1.39)
18/420 0.61 (0.33 to 1.13)
0 1 2 2.5
SHR (95% CI)
Musculoskeletal
SEP indicator n(cases)/n(total) SHR (95% CI)
Education
High 17/190 1.00 (Ref.)
Intermediate 75/672 1.38 (0.82 to 2.33)
Low 57/704 1.15 (0.67 to 1.98)
Occupation
High 50/475 1.00 (Ref.)
Intermediate 56/589 1.08 (0.73 to 1.60)
Low 43/502 1.00 (0.65 to 1.53)
Source of income
High 63/695 1.00 (Ref.)
Intermediate 34/262 1.40 (0.92 to 2.12)
Low 52/609 1.17 (0.80 to 1.72)
Cumulative SEP
High (0–2) 54/520 1.00 (Ref.)
Intermediate (3–4) 58/626 1.04 (0.71 to 1.53)
Low (5–6) 37/420 1.16 (0.74 to 1.83)
0 1 2 2.5
SHR (95% CI)
Neoplasm
n(cases)/n(total) SHR (95% CI)
26/190 1.00 (Ref.)
93/672 1.06 (0.69 to 1.63)
93/704 1.10 (0.71 to 1.70)
74/475 1.00 (Ref.)
66/589 0.77 (0.55 to 1.08)
72/502 1.03 (0.74 to 1.43)
101/695 1.00 (Ref.)
29/262 0.74 (0.49 to 1.12)
82/609 1.06 (0.79 to 1.44)
77/520 1.00 (Ref.)
77/626 0.89 (0.65 to 1.23)
58/420 1.12 (0.78 to 1.60)
0 1 2 2.5
SHR (95% CI)
Other
n(cases)/n(total) SHR (95% CI)
36/190 1.00 (Ref.)
129/672 1.03 (0.71 to 1.48)
121/704 0.95 (0.65 to 1.38)
83/475 1.00 (Ref.)
104/589 1.05 (0.78 to 1.40)
99/502 1.22 (0.90 to 1.65)
127/695 1.00 (Ref.)
53/262 1.12 (0.81 to 1.55)
106/609 1.00 (0.77 to 1.31)
95/520 1.00 (Ref.)
116/626 1.04 (0.79 to 1.37)
75/420 1.07 (0.77 to 1.48)
0 1 2 2.5
SHR (95% CI)

© 2022 X

eFigure 3. Association of socioeconomic position indicators with first cause-specific elective hospitalization, CHAMP.

Abbreviations: SEP, socioeconomic position; SHR, sub-hazard ratio. N = 1566. We used calendar year as the time scale, with survivors having a censoring date of 31 December 2017 for elective hospitalization due to circulatory (person-years follow-up = 13074), digestive (person-years follow-up = 13252), genitourinary (person-years follow-up = 13664), musculoskeletal (person-years follow-up = 13169), neoplasm (person-years follow-up = 13283) and other diseases (person-years follow-up = 12771). All estimates were adjusted for age, age squared, country of birth and marital status.

© 2022 Xu P et al. *JAMA Network Open.*

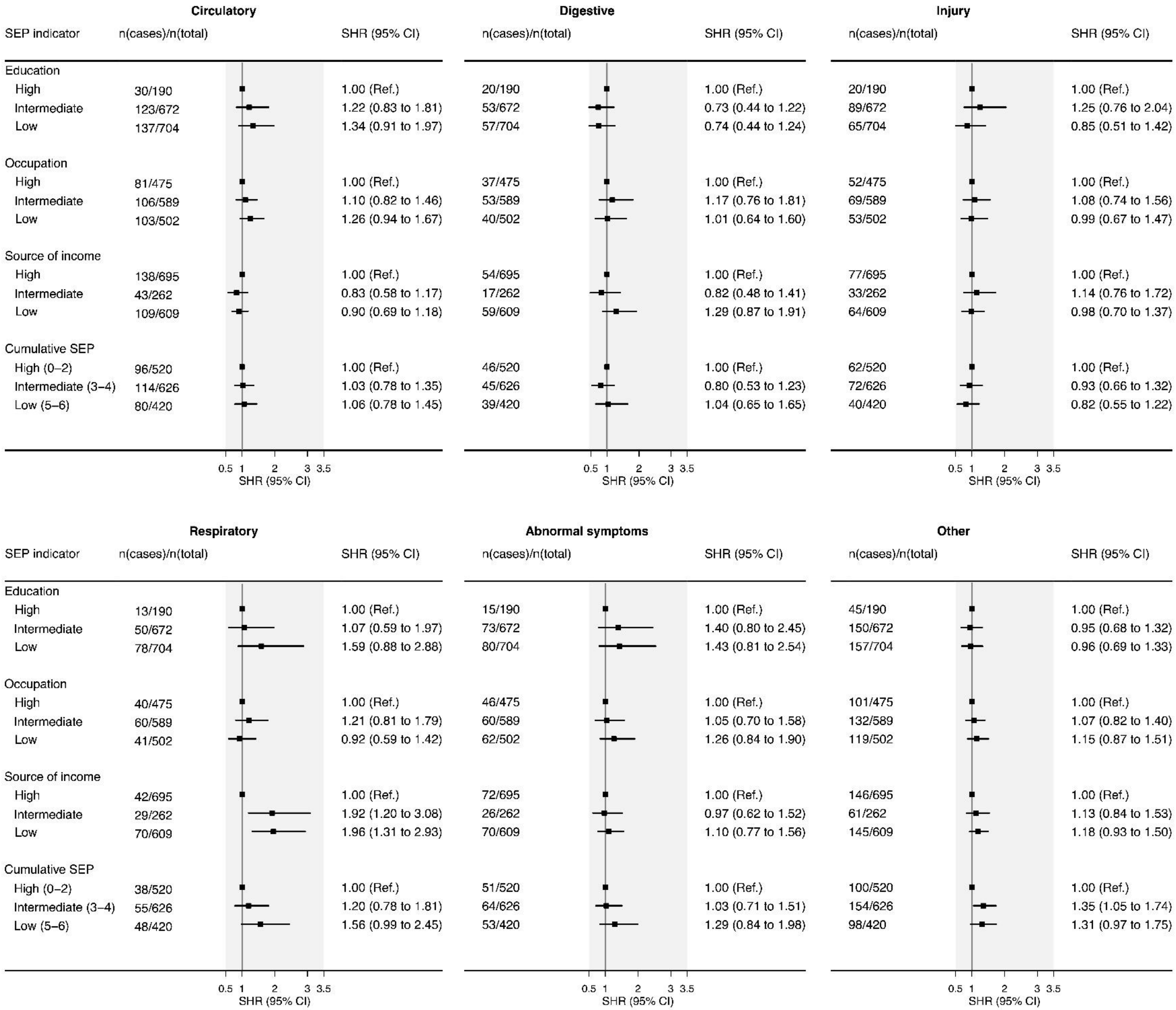
Circulatory
SEP indicator
n(cases)/n(total)
SHR (95% CI)
Education
High 30/190 1.00 (Ref.)
Intermediate 123/672 1.22 (0.83 to 1.81)
Low 137/704 1.34 (0.91 to 1.97)
Occupation
High 81/475 1.00 (Ref.)
Intermediate 106/589 1.10 (0.82 to 1.46)
Low 103/502 1.26 (0.94 to 1.67)
Source of income
High 138/695 1.00 (Ref.)
Intermediate 43/262 0.83 (0.58 to 1.17)
Low 109/609 0.90 (0.69 to 1.18)
Cumulative SEP
High (0–2) 96/520 1.00 (Ref.)
Intermediate (3–4) 114/626 1.03 (0.78 to 1.35)
Low (5–6) 80/420 1.06 (0.78 to 1.45)
0.5 1 2 3 3.5
SHR (95% CI)
Digestive
n(cases)/n(total)
SHR (95% CI)
20/190 1.00 (Ref.)
53/672 0.73 (0.44 to 1.22)
57/704 0.74 (0.44 to 1.24)
37/475 1.00 (Ref.)
53/589 1.17 (0.76 to 1.81)
40/502 1.01 (0.64 to 1.60)
54/695 1.00 (Ref.)
17/262 0.82 (0.48 to 1.41)
59/609 1.29 (0.87 to 1.91)
46/520 1.00 (Ref.)
45/626 0.80 (0.53 to 1.23)
39/420 1.04 (0.65 to 1.65)
0.5 1 2 3 3.5
SHR (95% CI)
Injury
n(cases)/n(total)
SHR (95% CI)
20/190 1.00 (Ref.)
89/672 1.25 (0.76 to 2.04)
65/704 0.85 (0.51 to 1.42)
52/475 1.00 (Ref.)
69/589 1.08 (0.74 to 1.56)
53/502 0.99 (0.67 to 1.47)
77/695 1.00 (Ref.)
33/262 1.14 (0.76 to 1.72)
64/609 0.98 (0.70 to 1.37)
62/520 1.00 (Ref.)
72/626 0.93 (0.66 to 1.32)
40/420 0.82 (0.55 to 1.22)
0.5 1 2 3 3.5
SHR (95% CI)
Respiratory
SEP indicator
n(cases)/n(total)
SHR (95% CI)
Education
High 13/190 1.00 (Ref.)
Intermediate 50/672 1.07 (0.59 to 1.97)
Low 78/704 1.59 (0.88 to 2.88)
Occupation
High 40/475 1.00 (Ref.)
Intermediate 60/589 1.21 (0.81 to 1.79)
Low 41/502 0.92 (0.59 to 1.42)
Source of income
High 42/695 1.00 (Ref.)
Intermediate 29/262 1.92 (1.20 to 3.08)
Low 70/609 1.96 (1.31 to 2.93)
Cumulative SEP
High (0–2) 38/520 1.00 (Ref.)
Intermediate (3–4) 55/626 1.20 (0.78 to 1.81)
Low (5–6) 48/420 1.56 (0.99 to 2.45)
0.5 1 2 3 3.5
SHR (95% CI)
Abnormal symptoms
n(cases)/n(total)
SHR (95% CI)
15/190 1.00 (Ref.)
73/672 1.40 (0.80 to 2.45)
80/704 1.43 (0.81 to 2.54)
46/475 1.00 (Ref.)
60/589 1.05 (0.70 to 1.58)
62/502 1.26 (0.84 to 1.90)
72/695 1.00 (Ref.)
26/262 0.97 (0.62 to 1.52)
70/609 1.10 (0.77 to 1.56)
51/520 1.00 (Ref.)
64/626 1.03 (0.71 to 1.51)
53/420 1.29 (0.84 to 1.98)
0.5 1 2 3 3.5
SHR (95% CI)
Other
n(cases)/n(total)
SHR (95% CI)
45/190 1.00 (Ref.)
150/672 0.95 (0.68 to 1.32)
157/704 0.96 (0.69 to 1.33)
101/475 1.00 (Ref.)
132/589 1.07 (0.82 to 1.40)
119/502 1.15 (0.87 to 1.51)
146/695 1.00 (Ref.)
61/262 1.13 (0.84 to 1.53)
145/609 1.18 (0.93 to 1.50)
100/520 1.00 (Ref.)
154/626 1.35 (1.05 to 1.74)
98/420 1.31 (0.97 to 1.75)
0.5 1 2 3 3.5
SHR (95% CI)

© 2022 X

eFigure 4. Association of socioeconomic position indicators with first cause-specific non-elective hospitalization, CHAMP.

Abbreviations: SEP, socioeconomic position; SHR, sub-hazard ratio. N = 1566. We used calendar year as the time scale, with survivors having a censoring date of 31 December 2017 for elective hospitalization due to circulatory (person-years follow-up = 12703), digestive (person-years follow-up = 13428), injury (person-years follow-up = 13449), respiratory (person-years follow-up = 13631), abnormal symptoms (person-years follow-up = 13332) and other diseases (person-years follow-up = 12762). All estimates were adjusted for age, age squared, country of birth and marital status.

© 2022 Xu P et al. *JAMA Network Open.*
